# Efficient and scalable synthesis of 1,5-diamino-2-hydroxy-pentane from l-lysine via cascade catalysis using engineered *Escherichia coli*

**DOI:** 10.1186/s12934-022-01864-8

**Published:** 2022-07-16

**Authors:** Yangyang Li, Alei Zhang, Shewei Hu, Kequan Chen, Pingkai Ouyang

**Affiliations:** 1 College of Biotechnology and Pharmaceutical Engineering, Nanjing Tech University, Nanjing, 211816 China; 2 State Key Laboratory of Materials-Oriented Chemical Engineering, Nanjing Tech University, Nanjing, 211816 China

**Keywords:** 1,5-diamino-2-hydroxy-pentane, (2S, 3S)-3-hydroxylysine, l-lysine transporters, Whole-cell biocatalyst, l-lysine hydroxylase, l-lysine decarboxylase, Scalable synthesis

## Abstract

**Background:**

1,5-Diamino-2-hydroxy-pentane (2-OH-PDA), as a new type of aliphatic amino alcohol, has potential applications in the pharmaceutical, chemical, and materials industries. Currently, 2-OH-PDA production has only been realized via pure enzyme catalysis from lysine hydroxylation and decarboxylation, which faces great challenges for scale-up production. However, the use of a cell factory is very promising for the production of 2-OH-PDA for industrial applications, but the substrate transport rate, appropriate catalytic environment (pH, temperature, ions) and separation method restrict its efficient synthesis. Here, a strategy was developed to produce 2-OH-PDA via an efficient, green and sustainable biosynthetic method on an industrial scale.

**Results:**

In this study, an approach was created for efficient 2-OH-PDA production from l-lysine using engineered *E. coli* BL21 (DE3) cell catalysis by a two-stage hydroxylation and decarboxylation process. In the hydroxylation stage, strain B14 coexpressing l-lysine 3-hydroxylase K3H and the lysine transporter CadB-argT enhanced the biosynthesis of (2S,3S)-3-hydroxylysine (hydroxylysine) compared with strain B1 overexpressing K3H. The titre of hydroxylysine synthesized by B14 was 2.1 times higher than that synthesized by B1. Then, in the decarboxylation stage, CadA showed the highest hydroxylysine activity among the four decarboxylases investigated. Based on the results from three feeding strategies, l-lysine was employed to produce 110.5 g/L hydroxylysine, which was subsequently decarboxylated to generate a 2-OH-PDA titre of 80.5 g/L with 62.6% molar yield in a 5-L fermenter. In addition, 2-OH-PDA with 95.6% purity was obtained by solid-phase extraction. Thus, the proposed two-stage whole-cell biocatalysis approach is a green and effective method for producing 2-OH-PDA on an industrial scale.

**Conclusions:**

The whole-cell catalytic system showed a sufficiently high capability to convert lysine into 2-OH-PDA. Furthermore, the high titre of 2-OH-PDA is conducive to separation and possesses the prospect of industrial scale production by whole-cell catalysis.

**Supplementary Information:**

The online version contains supplementary material available at 10.1186/s12934-022-01864-8.

## Background

Chiral amino alcohols are important chemical intermediates that have been applied in many fields because they possess active nucleophilic groups (–NH_3_, –OH) [1]. For instance, amino alcohols can string together chemical building blocks for synthesizing sphingolipids [2], antibiotics [3], and antiviral glycosidase inhibitors [4], as well as herbicides and insecticides [5]. In addition, amino alcohols can also be used in epoxy resin curing agents [6], corrosion inhibitors [7], and carbon dioxide absorbents, based on their properties of low toxicity [8], room temperature curing [9], low viscosity [10], and easy ductility [11].

1,5-Diamino-2-hydroxy-pentane (2-OH-PDA), a new kind of aliphatic amino alcohol, has potential pharmaceutical [12], material [13], agrochemical [14], surfactant and energy applications [15]. 2-OH-PDA can be biosynthesized from l-lysine via enzymatic hydroxylation and decarboxylation [16]. As a primary microbial metabolite, l-lysine can be efficiently produced via fermentation and the ‘‘economy of scale”, while only compensating for the falling market prices [17, 18]. Therefore, the conversion of l-lysine to produce high-value 2-OH-PDA is of great economic value. Currently, several dioxygenases, such as lysine hydroxylase, have been reported to catalyse the conversion of l-lysine into hydroxylysine [19–21]. However, only one study reported the direct biosynthesis of 2-OH-PDA from l-lysine by cascading a lysine hydroxylase and a pyridoxal phosphate-dependent decarboxylase using purified enzymes for the process [16]. Compared to the synthesis of 2-OH-PDA using purified enzymes, whole-cell catalysis can be more efficient, as intracellular enzymes are more stable [22]. A study found that the cost of whole-cell catalysts is 10% of that of pure enzymes based on the cost contribution from biocatalyst production to biocatalytic processes [23]. In addition, the subsequent separation/purification of 2-OH-PDA would be simpler than that with direct enzyme catalysis [24]. For example, a whole-cell system catalysed high-level l-lysine to produce 5-aminovalerate with a titre of 90.59 g/L in 94.2% molar yield [25]. Thus, the establishment of similar efficient processes for 2-OH-PDA preparation is of great importance.

**Scheme 1:**
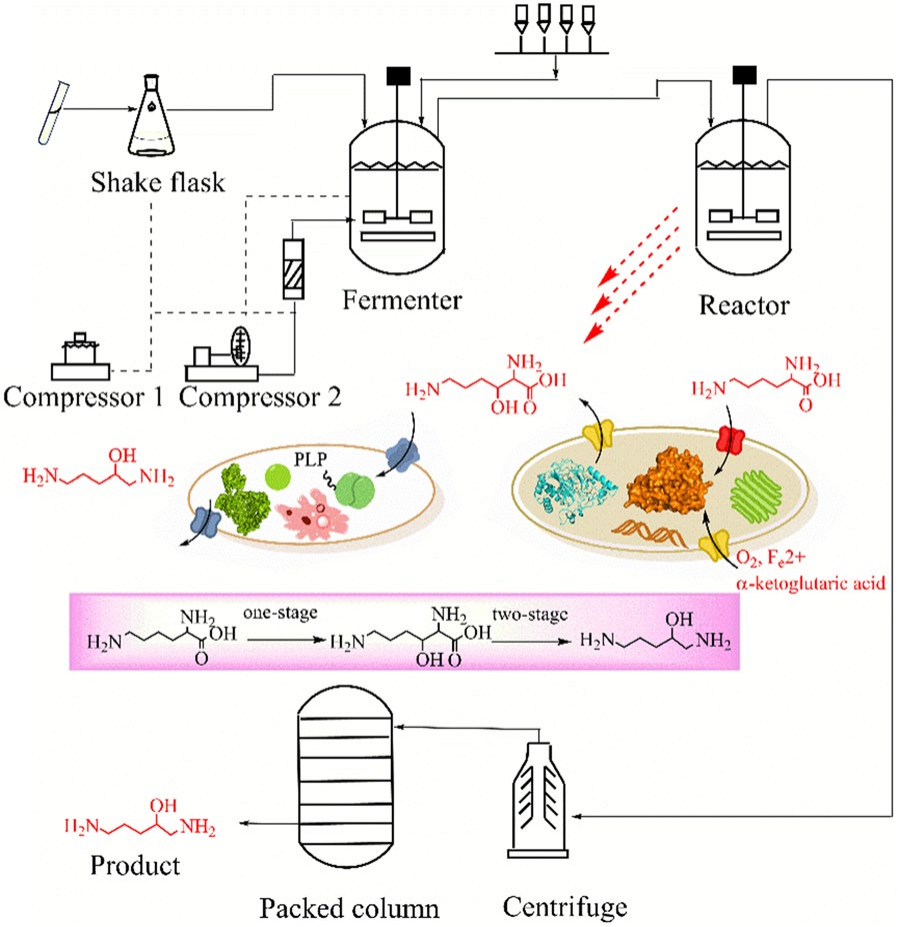
Reaction process

This study proposed the efficient synthesis of 2-OH-PDA from l-lysine via a two-stage biocatalytic process using whole engineered *E. coli* BL21 (DE3) cells for the first time. The effect of related lysine transporters on the production of the intermediate hydroxylysine was evaluated. Furthermore, the hydroxylation and decarboxylation conditions for converting l-lysine into 2-OH-PDA were optimized. In addition, the fed-batch production of 2-OH-PDA in a 5 L reactor via whole-cell biotransformation was studied, followed by isolation and purification of the 2-OH-PDA product (Scheme 1).

## Results and discussion

### Comparison of the one-stage and two-stage syntheses of 1,5-diamino-2-hydroxy-pentane using engineered *E. coli* whole cells

l-Lysine decarboxylase can act on both l-lysine and hydroxylysine [16, 20, 26]. Therefore, 2-OH-PDA was synthesized using both one-stage and two-stage methods. In the one-stage protocol, the engineered *E. coli* strains B1 (pRSFDuet1-*K3H*), which overexpresses l-lysine hydroxylase, and B15 (pRSFDuet1-*CadA*), which overexpresses l-lysine hydroxylase/hydroxylysine decarboxylase [27], were mixed for the conversion of l-lysine. Gradually increasing the B15 concentration to 0.5 g (DCW)/L (dry cell weight) while maintaining B1 at 5 g (DCW)/L resulted in an extremely low titre of 2-OH-PDA, despite the complete consumption of l-lysine and the nondetection of hydroxylysine (Table 1, one-stage). When the B15 concentration was raised to 5 g (DCW)/L, no 2-OH-PDA was found. This was probably caused by the preference of lysine decarboxylase for l-lysine as its substrate. Yamamoto also found that lysine decarboxylase was less active with δ-hydroxylysine than l-lysine [27].

**Table 1:** Comparison the one- and two-stage syntheses of 2-OH-PDA by engineered *E. coli*

Strategy	B1 (g/L DCW)	B15 (g/L DCW)	l-lysine (g/L)	Hydroxylysine (g/L)	2-OH-PDA (g/L)
One-stage	5	0	19.8 ± 0.3	12.1 ± 0.6	0
	5	0.5	0	0	0.8 ± 0.1
	5	2.5	0	0	0.1 ± 0.04
	5	5	0	0	0
Two-stage	5	5	0	0	9.3 ± 0.7

B1 (pRSFDuet1-*K3H*, expressing hydroxylase) and B15 (pRSFDuet1-*CadA*, expressing decarboxylase) were simultaneously added at different concentrations to the reaction mixture simultaneously for the one-stage synthesis. In the two-stage process, B1 hydroxylated l-lysine to produce (2S,3S)-3-hydroxylysine for 24 h, and then B15 was added to decarboxylate hydroxylysine to produce 2-OH-PDA over the next 24 h

For the two-stage synthesis strategy, when the concentration of hydroxylysine was not increased by catalysis by strain B1 (Additional file 1: Fig. S4), strain B15 was added to the reaction system, followed by the synthesis of 2-OH-PDA. This approach obtained a titre of 9.3 g/L 2-OH-PDA (Table 1); therefore, two-stage synthesis was selected for further optimization.

### Effect of lysine transporters on (2S,3S)-3-hydroxylysine production

**Fig. 1:**
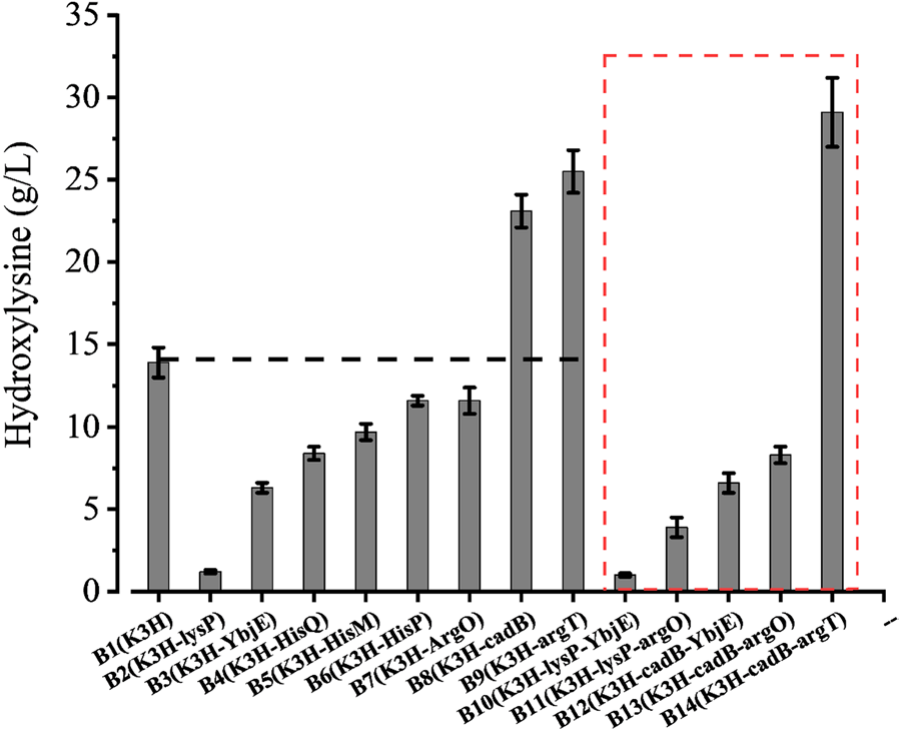
Effect of lysine transporters on hydroxylysine production. The reaction for hydroxylysine synthesis contained 30 g/L l-lysine, 36 g/L α-ketoglutaric acid, 5 mM Vc, 5 mM FeSO_4_, and 5 g dry cell weight (DCW)/L and was incubated at 30 °C with shaking at 200 rpm for 30 h. Overexpression of lysine hydroxylase: strain B1 (pRSFDuet1-*K3H*). Coexpression of lysine hydroxylase and a single lysine transporter protein: strains B2 (pRSFDuet1-*K3H-lysP*), B3 (pRSFDuet1-*K3H-YbjE*), B4 (pRSFDuet1-*K3H-HisQ*), B5 (pRSFDuet1-*K3H-HisM*), B6 (pRSFDuet1-*K3H-HisP*), B7 (pRSFDuet1-*K3H-argO*), B8 (pRSFDuet1-*K3H-cadB*), and B9 (pRSFDuet1-*K3H-argT*). Coexpression of lysine hydroxylase and combined lysine transporter proteins: strains B10 (pRSFDuet1-*K3H*-*lysP*-*YbjE*), B11 (pRSFDuet1-*K3H*-*lysP*-*argO*), B12 (pRSFDuet1-*K3H*-*CadB*-*YbjE*), B13 (pRSFDuet1-*K3H*-*CadB*-*argO*), and B14 (pRSFDuet1-*K3H*-*CadB*-*argT*)

Amino acid transporters may serve to improve substrate transport for cellular metabolism and catalysis [28]. Ma reported that overexpression of a lysine/cadaverine antiporter in *E. coli* can increase cadaverine production during whole-cell catalysis [29]. Thus, the effect of lysine transporters on hydroxylysine production was investigated via their single and joint overexpression in *E. coli* BL21 (DE3). As shown in Fig. 1, the hydroxylysine titres from B8 (pRSFDuet1-*K3H*-*CadB*) and B9 (pRSFDuet1-*K3H*-*argT*) cells were 23.1 ± 1.0 and 25.5 ± 1.3 g/L, respectively, which were 1.6 and 1.8 times that of B1 (pRSFDuet1-*K3H*) (13.9 ± 0.9 g/L). However, hydroxylysine production by the other strains, B2 (pRSFDuet1-*K3H-lysP*) (1.2 ± 0.1 g/L), B3 (pRSFDuet1-*K3H-YbjE*) (6.3 ± 0.3 g/L), B4 (pRSFDuet1-*K3H-HisQ*) (8.4 ± 0.4 g/L), B5 (pRSFDuet1-*K3H-HisM*) (9.7 ± 0.5 g/L), B6 (pRSFDuet1-*K3H-HisP*) (11.6 ± 0.3 g/L), and B7 (pRSFDuet1-*K3H-argO*) (11.6 ± 0.8 g/L), was lower than that by B1 (Fig. 1).

The transporters HisM, HisP, HisQ, ArgT, CadB, and LysP are responsible for l-lysine absorption, while Argo and YbjE are involved in the secretion of l-lysine [30]. In the ABC transporter superfamily, HisP hydrolyses ATP to change the transmembrane structure of HisM and HisQ, which complete l-lysine transport. Insufficient ATP supply in resting cells and low membrane protein expression may therefore reduce free cell catalysis product yield [31]. This may be the reason for the poor hydroxylysine production from B4, B5, and B6 cells compared to that from B1 cells. Under aerobic conditions at pH ≥ 6.0, the transport capacity of the permease LysP is less than 3 nmol of L-lysine/mg pure protein [32], which can explain the poor titre from the B2 cells.

However, the resting cells of B8 and B9 exhibited higher activity than those of B1. B8 and B9 cells express other transporters that function differently from HisP: ArgT recruits substrates and accelerates substrate uptake by cells, while CadB, a lysine/cadaverine antiporter, facilitates cadaverine excretion and coordinates lysine uptake, driven solely by the concentration gradient without other energy inputs [29]. This indicated that the ArgT and CadB transporters functioned better in lysine transport and were more suited for hydroxylysine production.

Subsequently, l-lysine uptake and secretion were combined by jointly expressing both types of transporter proteins. B14 (pRSFDuet1-*K3H*-*CadB*-*argT*) produced 1.3- and 1.1-fold more hydroxylysine than B8 and B9, respectively. In contrast, B10 (pRSFDuet1-*K3H*-*lysP*-*YbjE*), B11 (pRSFDuet1-*K3H*-*lysP*-*argO*), B12 (pRSFDuet1-*K3H*-*CadB*-*YbjE*), and B13 (pRSFDuet1-*K3H*-*CadB*-*argO*) produced low levels of hydroxylysine (6.6 ± 0.6, 8.3 ± 0.5, 1.0 ± 0.1, and 3.9 ± 0.6 g/L, respectively). Thus, strain B14 was used for hydroxylysine production.

### Optimization of (2S,3S)-3-hydroxylysine production

**Fig. 2:**
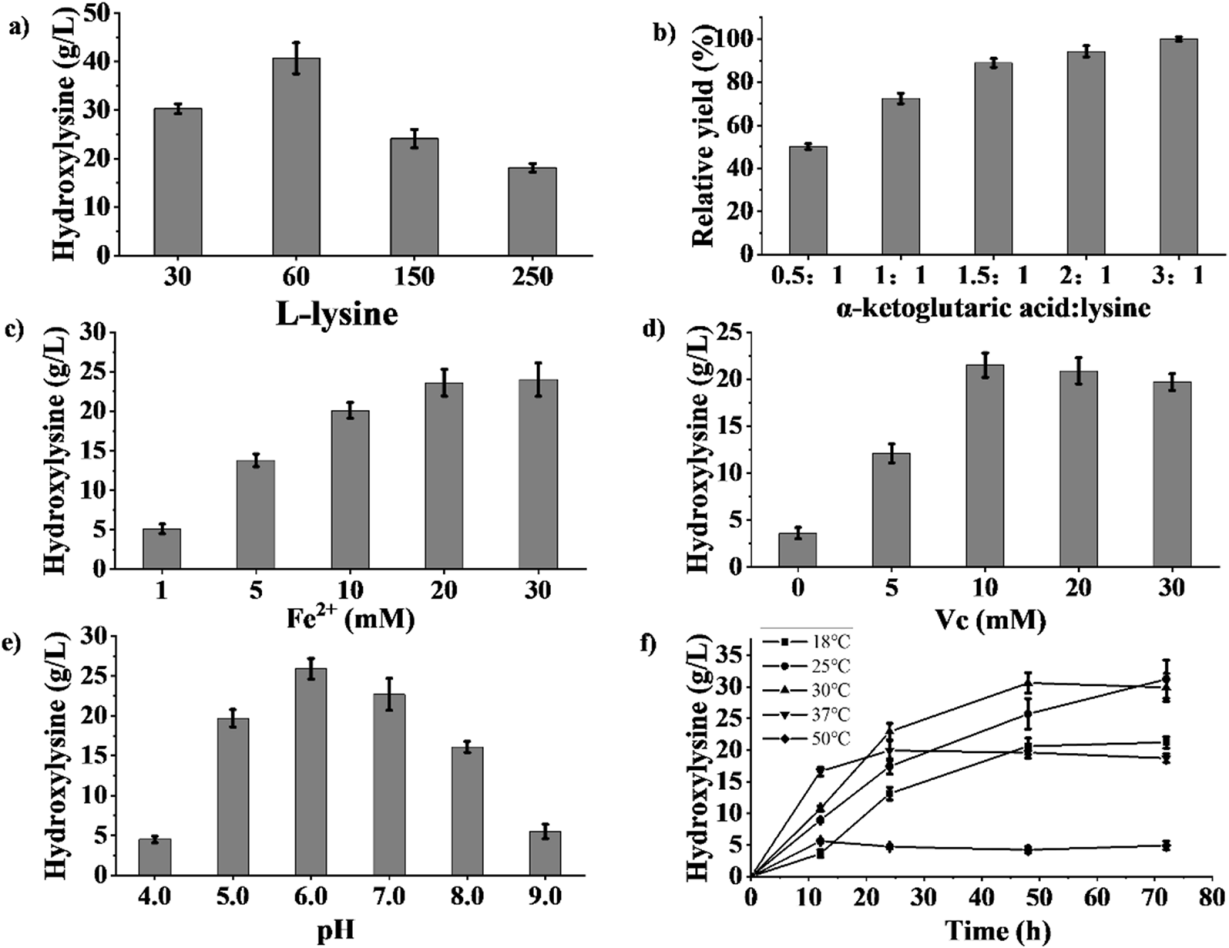
Effects of l-lysine concentration (**a**), ratio of α-ketoglutarate acid/lysine (**b**), concentrations of Fe(II) **c** and Vc (**d**), pH (**e**), and temperature **f** on hydroxylysine synthesis. Washed cells were resuspended in 20 mL of 50 mM PBS at varying pH values (4.0, 5.0, 6.0, 7.0, 8.0, and 9.0). The hydroxylysine synthesis reaction contained l-lysine (30, 60, 150, and 250 g/L), α-ketoglutaric acid (molar ratio, 0.5:1, 1:1, 1.5:1, 2:1, and 3:1), Vc (0, 5, 10, 20, and 30 mM), FeSO_4_ (1, 5, 10, 20, and 30 mM), and 5 g dry cell weight (DCW)/L and was incubated at different temperatures (18, 25, 30, 37, and 50 °C) with shaking at 200 rpm for different lengths of time (0, 12, 24, 48, and 72 h)

In whole-cell catalysis, enzymatic activity may be influenced by the substrate concentration [33]. Thus, the l-lysine concentration was varied to test its effect on hydroxylysine production. As shown in Fig. 2a, hydroxylysine production and the l-lysine conversion titre both decreased at l-lysine concentrations > 150 g/L. This was probably caused by the inhibition of l-lysine uptake during whole-cell catalysis due to reduced transporter protein activity towards the higher concentration of substrate [29]. In addition, the cosubstrate α-ketoglutaric acid activates oxygen molecules and participates in hydroxylase catalysis [34]. Thus, the amount of α-ketoglutarate acid directly influences the catalytic efficiency of Fe(II)/α-ketoglutarate-dependent dioxygenases. Several studies have constructed chassis strains to generate α-ketoglutarate acid and facilitate Fe(II)/α-ketoglutarate-dependent dioxygenase-mediated C–H bond oxidation [35–37]. Here, the effect of the α-ketoglutarate/lysine ratio on *E. coli* whole-cell catalysis was evaluated. The hydroxylysine yield increased with increasing α-ketoglutarate/lysine ratio (Fig. 2b), as α-ketoglutarate was gradually degraded into succinic acid and carbon dioxide [38], and a higher α-ketoglutarate dosage was conducive to the reaction. When the α-ketoglutarate/lysine ratio exceeded 1.5:1, a hydroxylysine mass concentration of 5.5% was achieved, which was 11.1% higher than that observed in the control. It was previously reported that increasing the molar ratio of ketoglutarate/lysine is conducive to producing cis-3-hydroxypipecolic acid by whole-cell catalysis [39].

The cofactor Fe(II) assists hydroxylases when acting on structurally diverse substrates and triggers the process of hydroxylation [34]. The effect of Fe(II) concentration on producing hydroxylysine was therefore investigated. As seen in Fig. 2c, the hydroxylysine titre increased with an increase in Fe(II) concentration from 5.1 ± 0.6 g/L in the presence of 1 mM Fe(II) to 20.1 ± 1 g/L with 10 mM Fe(II), after which the increasing trend slowed down at higher Fe(II) concentrations. l-Ascorbic acid (Vc) is a reductant that promotes C–H oxidation catalysed by Fe(II)/α-ketoglutarate-dependent dioxygenases [40]. Under aerobic conditions, it is possible that Vc acts in part to retain free Fe(II) in its reduced form and keeps the enzyme or enzyme·Fe(II) complex in a reduced state in solution between catalytic cycles [41]. Adding Vc to the reaction led to a considerable increase in the accumulation of hydroxylysine after 24 h (Fig. 2d). However, Vc concentrations exceeding 10 mM led to a slight decrease in the whole-cell catalytic activity and hydroxylysine production.

pH affects enzyme catalytic activity and cell stability, and whole-cell catalytic activity can be enhanced using a suitable buffer [20]. The influence of pH on whole-cell catalysis for hydroxylysine production was then evaluated (Fig. 2e). The hydroxylysine titre reached a maximum of 25.9 ± 1.3 g/L with an initial L-lysine concentration of 30 g/L at the optimum pH of 6.0, with a relatively high titre (22.7 ± 2 g/L) achieved at pH 7. However, l-lysine hydroxylase activity was considerably adversely affected under acidic (pH < 5.0, 4.5 ± 0.4 g/L) or alkaline (pH > 8.0, 5.5 ± 0.9 g/L) conditions.

Subsequently, the influence of temperature on whole-cell catalysis was evaluated. As seen in Fig. 2f, gradual accumulation of hydroxylysine was observed at different temperatures, except at 50 °C, where the hydroxylase showed poor activity. The maximum hydroxylysine concentration after 12 h was detected at 37 °C (16.6 ± 0.7 g/L), while the maximum after 48 h was detected at 30 °C (30.6 ± 1.6 g/L). At these temperatures, hydroxylysine production quickly reached an equilibrium, whereas the whole-cell catalytic reaction required ≥ 72 h to reach equilibrium at 18 °C and 25 °C.

### Optimization of the 1,5-diamino-2-hydroxy-pentane generation module

**Fig. 3:**
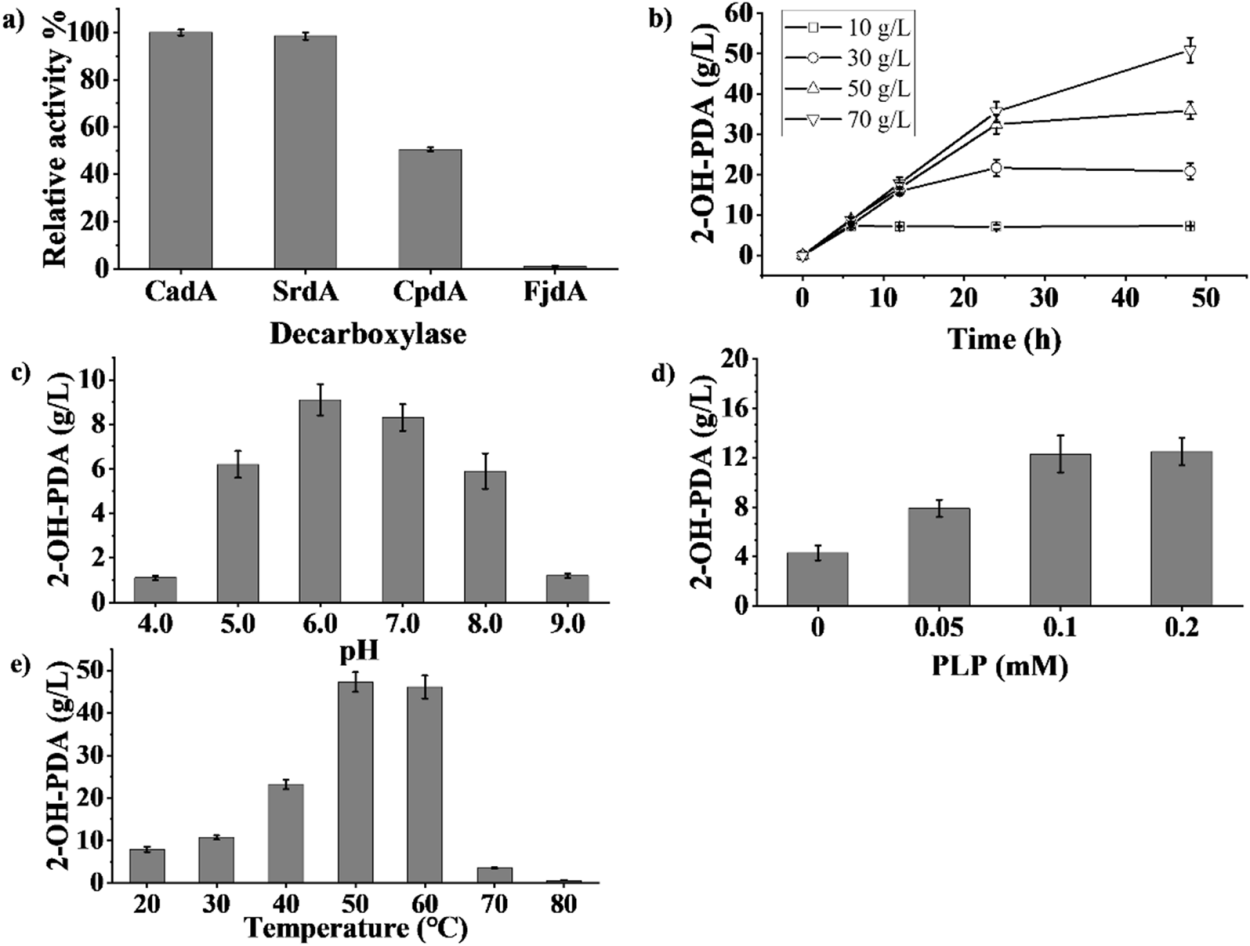
Effects of decarboxylase (**a**), l-lysine concentration (**b**), pH (**c**), PLP concentration (**d**), and temperature **e** on 2-OH-PDA titre. The 2-OH-PDA synthesis reaction contained hydroxylysine (10, 30, 50, and 70 g/L), PLP (0, 0.05, 0.1, and 0.2 mM), and 5 g DCW/L and was incubated at different pH values (4.0, 5.0, 6.0, 7.0, 8.0, and 9.0) and temperatures (20, 30, 40, 50, 60, 70, and 80 °C) with shaking at 200 rpm for different lengths of time (0, 6, 12, 24, and 48 h). Samples (0.3 mL) were collected every few hours

To enable the efficient decarboxylation of hydroxylysine to 2-OH-PDA, decarboxylases CadA from *E. coli* BL21 (DE3), SrdA from *Selenomonas ruminantium*, CpdA from *Chitinophaga pinensis*, and FjdA from *Flavobacterium johnsoniae* were screened. The results (Fig. 3a) indicated that the relative catalytic activities were 100% ± 2.2% (CadA), 98.5 ± 2.2% (SrdA), 50.6 ± 0.8% (CpdA), and 1.1 ± 0.2% (FjdA). Therefore, CadA was selected for the second stage of the whole-cell catalysis to generate 2-OH-PDA and further study hydroxylysine decarboxylation.

In this study, whole-cell biocatalysis was performed with the engineered B15 (pRSFDuet1-*CadA*) strain at 5 g/L DCW. As shown in Fig. 3b, the maximum concentration of 2-OH-PDA reached 50.9 ± 1.6 g/L from 70 g/L hydroxylysine at 48 h. At concentrations lower than 70 g/L, hydroxylysine was completely converted to 2-OH-PDA after 24 h. Interestingly, whole-cell decarboxylation at different hydroxylysine concentrations proceeded at a similar rate until approximately 24 h, except at 10 g/L. Indeed, the reaction pH gradually increased and exceeded 8.0 when 70 g/L hydroxylysine was used (data not shown). The pH optimization results (Fig. 3c) showed similar titres of 2-OH-PDA at pH 6.0 (9.1 ± 0.9 g/L) and pH 7.0 (8.3 ± 0.8 g/L) after 6 h. The 2-OH-PDA concentration achieved at pH 5.0 (6.2 ± 0.6 g/L) was equal to that at pH 8.0. More extreme pH changes severely inhibited the whole-cell catalytic reaction, with 2-OH-PDA accumulation of only 1.1 ± 0.1 g/L at pH 4.0 and 1.2 ± 0.1 g/L at pH 9.0. Under acidic conditions (approximately pH 6.0), CadA maintains the ideal decamer conformation, and the dimer forming the decamer possesses the proper decarboxylase catalytic centre, while the polymerized structure at pH > 9.0 and depolymerized structure at pH < 5.0 do not effectively decarboxylate l-lysine [26].

Decarboxylases utilize PLP as a cofactor to decarboxylate the α-carbonyl group of their target amino acid in a reaction that consumes an intracellular proton [42]. Therefore, the effect of PLP concentration on decarboxylase activity was examined (Fig. 3d). The titre of 2-OH-PDA (4.3 ± 0.6 g/L) obtained without the addition of PLP was only 35.0% that obtained with 0.05 mM PLP (7.9 ± 0.7 g/L), suggesting that the amount of intracellular PLP was insufficient for whole-cell catalysis [43, 44]. Indeed, gradually increasing the PLP concentration to 0.1 mM increased the titre of 2-OH-PDA (to 12.3 ± 1.2 g/L), after which it remained stable. The 2-OH-PDA titre also improved with gradually increasing temperature from 20 to 60 °C (Fig. 3e), with similar 2-OH-PDA titres of 47.3 ± 2.3 and 46.1 ± 2.7 g/L at 50 and 60 °C, respectively. Some studies found that the optimal reaction temperature of CadA was approximately 55 °C and that similar activities were observed at 50 and 60 °C for whole-cell catalysis [45]. However, the product concentration drastically decreased at higher temperatures, with titres of 3.5 ± 0.1 g/L and 0.6 ± 0.05 g/L at 70 and 80 °C, respectively. Although CadA showed maximum activity at 50–60 °C, it showed low long-term thermal stability at temperatures exceeding 70 °C [46].

### Fed-batch production of 1,5-diamino-2-hydroxy-pentane in a 5-L fermenter

2-OH-PDA production was scaled up in a 5-L fermenter using the optimized reaction conditions. In the first stage, three l-lysine feeding strategies, as described in the Materials and Methods, were developed according to the l-lysine conversion rate to eliminate substrate inhibition. Strategy 3 afforded a hydroxylysine concentration of 110.5 ± 3.1 g/L at 65 h, which was 74.3 and 46.7% higher than those achieved using feed strategies 1 and 2, respectively (Table 2). Previous studies have reported an ~ 10 mM (1.62 g/L) titre of hydroxylysine using purified hydroxylases [20], which was enhanced to 86.1 g/L using an engineered hydroxylase in a 40 mL whole-cell catalysis reaction [21, 47]. However, the hydroxylysine titre achieved from the current study is higher than that obtained from both previous reports.

**Table 2:** Fed-batch transformation experiment using different lysine feeding strategies

Feed strategy	l- lysine (g/L)	Hydroxylysine (g/L)	2-OH-PDA (g/L)	Molar yield (hydroxylysine/l-lysine) (%)
1	150.1 ± 3.6	63.4 ± 3.6	46.2 ± 1.3	38.1 ± 1.1
2	170.8 ± 5.2	75.3 ± 2.5	54.9 ± 2.6	39.9 ± 1.9
3	159.6 ± 4.1	110.5 ± 5.1	80.5 ± 2.9	62.6 ± 2.3

l-Lysine was fed using three different strategies. (1) First, 150 g/L lysine and 180 g/L α-ketoglutaric acid were added at the start of the reaction. (2) l-Lysine was initially added at 60 g/L and α-ketoglutaric acid at 72 g/L. When the concentration of l-lysine dropped to 30 g/L, 60 g/L l-lysine and 72 g/L α-ketoglutaric acid were added to the reaction mixture. (3) A feeding rate of 2 g/(L·h) l-lysine and 2.4 g/(L·h) α-ketoglutaric acid was maintained throughout the reaction

Based on the hydroxylysine synthesis results, strain B15 (5 g/L DCW) and PLP were added to conduct the second stage of catalysis. The engineered strain showed good catalytic ability to completely convert hydroxylysine into 2-OH-PDA. The 2-OH-PDA titre reached 80.5 ± 2.1 g/L after 48 h, which is the highest titre reported to date. This result suggested that whole-cell transformation by recombinant *E. coli* is a feasible and highly effective approach for the large-scale production of 2-OH-PDA (Table 2) [48].

### Separation of the product 1,5-diamino-2-hydroxy-pentane

The 2-OH-PDA product was separated as described in the Experimental section. A recovery of 70.3% was achieved from the whole scaled-up separation of the transparent and yellowish liquid product. LC-Q-TOF–MS analysis of the product revealed a single peak at 19.577 min (Additional file 1: S4). The mass spectra of the product typically showed a single peak 341.1864 for the H^+^ adduct, which matched the molecular weight of 2-OH-PDA derivatized with Fmoc-Cl. Thus, the final isolated 2-OH-PDA product reached 95.6% purity.

## Conclusions

The current study offers a novel approach for efficiently synthetizing chiral amino alcohols by whole-cell catalysis and extended the multiple uses of lysine. Various lysine transporters and hydroxylysine decarboxylases were screened to improve 2-OH-PDA fabrication via whole-cell catalysis. Based on process optimization of hydroxylysine and 2-OH-PDA production, a fed-batch approach was adopted to enhance the bioconversion of l-lysine to 2-OH-PDA in a 5-L fermenter, in which the hydroxylysine and 2-OH-PDA titres reached 110.5 g/L and 80.5 g/L, respectively. The two-stage whole-cell catalysis approach is green and effective, which will enable efficient 2-OH-PDA production on an industrial scale.

## Materials and methods

### Chemicals

Tryptone and yeast extract were purchased from Oxoid Ltd. (Hampshire, UK). Unless otherwise stated, all other reagents were of analytical grade and purchased from Sinopharm Chemical Reagent Co., Ltd. (Shanghai, China). Other reagents and chemicals are described in the Additional file 1: Chemicals.

### Construction of the plasmids and strains

The bacterial strains and plasmids used in this study are summarized in Additional file 1: Table S1. The PCR primers used for plasmid construction are listed in Additional file 1: Table S2. DNA manipulations were performed according to standard protocols.

**Fig. 4:**
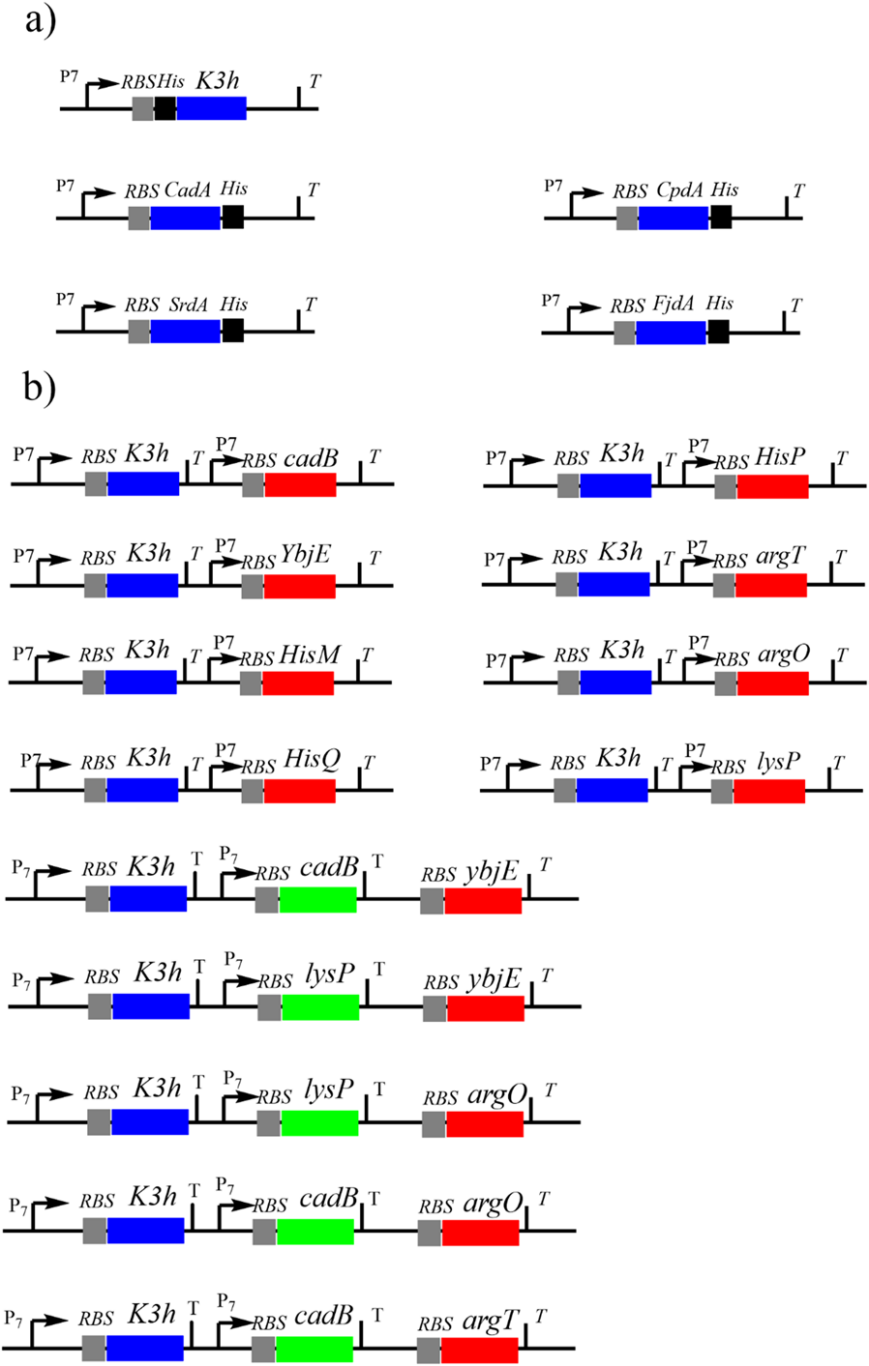
Schematic diagrams of the expression plasmids used in this study. **a** *K3H* is the lysine hydroxylase gene; *CadA, CpdA, SrdA* and *FjdA* are hydroxylysine decarboxylase genes. The blue bands are related genes. **b** The lysine hydroxylase and transporter genes were expressed in combination. The blue band is lysine hydroxylase (*K3H*), and the red and green bands are lysine transporter genes (*lysP*, *YbjE*, *HisQ*, *HisM*, *HisP*, *argO*, *cadB*, *argT*)

The codon-optimized gene encoding the l-lysine 3-hydroxylase K3H (GenBank: ABS05421.1) containing an N-terminal 6*His tag was introduced into the *Bam*H I and *Hin*d III sites of pRSFDuet1 to generate pRSFDuet1-*K3H* (strain B1)*.* Three genes encoding hydroxylysine decarboxylases CpdA (WP_012790490.1), SrdA (WP_014424780.1), and FjdA (WP_012025157.1), each containing a C-terminal 6*His tag, were introduced between the *Nco* I and *Not* I sites of pRSFDuet1 to generate pRSFDuet1-*CpdA* (strain B16), pRSFDuet1-*SrdA* (strain B17), and pRSFDuet1-*FjdA* (strain B18). These plasmids were synthesized by General Biol (Anhui, China). The gene encoding hydroxylysine decarboxylase CadA was amplified by PCR from *E. coli* BL21 (DE3) genomic DNA as the template (GenBank: AM946981.2) incorporating a C-terminal 6*His tag and cloned into the *Nco I* and *Not* I sites of pRSFDuet1, resulting in pRSFDuet1-*CadA* (Fig. 4a), that is, strain B15.

To construct the plasmids encoding lysine transporters (Fig. 4b), the *CadB* and *lysP* genes were separately amplified from *E. coli* BL21 (DE3) genomic DNA using the appropriate set of primers (Additional file 1: Table S2) and then inserted between the *Bgl* II and *Xho* I sites of pRSFDuet1-*K3H*. Additionally, the plasmids pRSFDuet1-*K3H*-*CadB*-*YbjE*, pRSFDuet1-*K3H*-*CadB*-*argO*, pRSFDuet1-*K3H*-*CadB*-*argT*, pRSFDuet1-*K3H*-*lysP*-*YbjE*, and pRSFDuet1-*K3H*-*lysP*-*argO* were constructed in the next two steps. For instance, to construct pRSFDuet1-*K3H*-*CadB*-*YbjE*, *CadB* and *YbjE* were fused by fusion PCR in two steps. In the first step, *CadB* was amplified using the primers CadB-F and CBYE-R from the template pRSFDuet1-*K3H*-*CadB*; similarly, the gene *YbjE* was amplified using the primers CBYE-F and YbjE-R from the template pRSFDuet1-*K3H*-*YbjE*. In the second step, the full-length gene fusion *CadB*-*YbjE* was assembled using overlap-extension PCR with the primers CadB-F and YbjE-R and the first step amplicons as templates for insertion into the *Bgl* II and *Xho* I sites of pRSFDuet1-*K3H*. More specifically, the restriction endonucleases *Eco*R I and *Eco*R V and RBS were added between *CadB* and *YbjE*. The relevant strains carrying lysine transporters were named strains B2 to B14. The engineered strains are listed in Additional file 1: Table S1.

### Expression and activity assay of hydroxylase and decarboxylase

The positive clones were cultivated in LB medium supplemented with kanamycin (50 µg/mL) at 37 °C and 200 rpm. When the OD_600_ of the culture reached 0.6–0.8, isopropyl-β-D- thiogalactopyranoside (IPTG) was added to a final concentration of 1.0 mM, and the temperature was lowered to 25 °C for 20­24 h. All recombinant protein samples were purified and analysed according to the description in the Additional file 1, Expression, purification and activity assay of hydroxylase and decarboxylase (Additional file 1: Figs. S1–S3).

### The effect of reaction conditions on whole-cell biocatalysis

The hydroxylation reaction of l-lysine by whole-cell catalyst was routinely performed in a 20 mL reaction volume. The effects of the concentration of l-lysine, Vc, FeSO_4_, substrate ratio (the ratio of l-lysine to α-ketoglutaric acid) and various temperatures were determined.

The decarboxylation of hydroxylysine for 2-OH-PDA synthesis by whole-cell catalysis was also performed in a 20 mL reaction volume. The effects of the concentration of hydroxylysine and PLP at various pH values and temperatures with shaking at 200 rpm were determined.

Additional file 1 regarding the conditions for whole-cell biocatalysis provides a detailed description.

### Fed-batch production of 1,5-diamino-2-hydroxy-pentane in a 5-L fermenter

The engineered *E. coli* strains were cultured and induced as described in the Additional file 1, High-density fermentation for the engineered strains with modifications [49]. Fed-batch bioconversion was conducted in a 5-L fermenter in a total reaction volume of 3 L at 30 °C and 300 rpm. The air speed was 3 L/min, and the pH was controlled at 6.0–7.0 using ammonia and phosphoric acid. The reaction mixture for (2S,3S)-3-hydroxylysine synthesis contained 10 mM Vc, 10 mM FeSO_4_, 5 g/L (DCW), 0.1% Triton X-100 and 50 mM PBS (pH 6.0). l-Lysine was fed using three different strategies. (1) 150 g/L lysine and 180 g/L α-ketoglutaric acid were added at the start of the reaction. (2) l-Lysine was initially added at 60 g/L and α-ketoglutaric acid at 72 g/L. When the concentration of l-lysine dropped to 30 g/L, 60 g/L l-lysine and 72 g/L α-ketoglutaric acid were added to the reaction mixture. (3) A feeding rate of 2 g/(L·h) l-lysine and 2.4 g/(L·h) α-ketoglutaric acid was maintained throughout the reaction.

When hydroxylysine synthesis reached equilibrium, the reaction mixture for 2-OH-PDA production was constructed. Briefly, 5 g/L (DCW) whole-cell biocatalyst and 0.1 mM PLP were also added to the hydroxylysine synthesis reaction, and the remaining conditions were maintained.

### Separation of 1,5-diamino-2-hydroxy-pentane

2-OH-PDA was isolated from the supernatant using two separation methods according to a previously reported method with modifications [48, 50]. To prepare the 2-OH-PDA standard, the reaction mixture was diluted with acidified water (4% H_3_PO_4_) in a 1:1 volume ratio. The diluted sample was loaded onto an SPE cartridge, which was then washed with 2% HCOOH in water, followed by MeOH. Elution was performed with a gradient of 4–32% NH_4_OH in MeOH. The solvent was evaporated under reduced pressure and dried, and the 2-OH-PDA obtained was used for HPLC detection (Additional file 1: Fig. S4 (b)).

### Analytical methods

The l-lysine, hydroxylysine, and 2-OH-PDA concentrations were determined according to a previous study with modifications [16, 39]. The reaction products were treated with 9-fluorenylmethoxycarbonyl chloride (Fmoc-Cl) using the precolumn derivatization method prior to HPLC analysis on an Agilent 1260 Infinity LC system (Agilent Technologies, Santa Clara, CA, USA) and liquid chromatography quadrupole time-of-flight mass spectrometry (LC-Q-TOF–MS) analysis (Additional file 1: Fig. S4). The specific steps are described in the Additional file 1: Analytical Methods. The product molar yield was calculated according to the following equation:

$$ {\text{Product yield }}\left( \% \right) \, = {\text{ product }}\left( {{\text{moles}}} \right)/{\text{substrate }}\left( {{\text{moles}}} \right). $$

## Supplementary Information

**Additional file 1: Table S1. **Strains and plasmids used in this work. **Table S2. **Primers used in this work. **Fig. S1.** Sodium dodecyl sulfate–polyacrylamide gel electrophoresis (SDS-PAGE) analysis showing expression l-lysine 3-hydroxylase K3H. **Fig. S2.** Sodium dodecyl sulfate–polyacrylamide gel electrophoresis (SDS-PAGE) analysis showing expression decarboxylases. **Fig. S3.** The expression of K3H (**a**), FjdA and CpdA (**b**), SrdA and CadA **c** in western blot analysis. **Fig. S4.** Hydroxylysine **a** and 2-OH-PDA **b** by LC-Q-TOF-MS analysis.

## Data Availability

All data generated or analysed during this study are included in this article and Additional file 1.

## Abbreviations

2-OH-PDA: 1,5-Diamino-2-hydroxy-pentane
Hydroxylysine: (2S, 3S)-3-hydroxylysine
K3H: l-lysine 3-hydroxylase
CpdA: Hydroxylysine decarboxylase,
SrdA: Hydroxylysine decarboxylase
FjdA: Hydroxylysine decarboxylase
CadA: Hydroxylysine decarboxylase
PLP: Pyridoxal-5'-phosphate monohydrate
PCR: Polymerase chain reaction
Vc: l-ascorbic acid
IPTG: Isopropyl-β-D- thiogalactopyranoside
DCW: Dry cell weight
Fmoc-Cl: 9-Fluorenylmethoxycarbonyl chloride
