# Supplementary material for: Efficient and scalable synthesis of 1,5-diamino-2-hydroxy-pentane from l-lysine via cascade catalysis using engineered Escherichia coli
Source: Microb Cell Fact. 2022 Jul 16;21:142. doi: 10.1186/s12934-022-01864-8 (PMC9288024; doi:10.1186/s12934-022-01864-8)
Supplement: Supplementary file 1 — Additional file 1: Table S1. Strains and plasmids used in this work. Table S2. Primers used in this work. Fig. S1. Sodium dodecyl sulfate–polyacrylamide gel electrophoresis (SDS-PAGE) analysis showing expression l-lysine 3-hydroxylase K3H. Fig. S2. Sodium dodecyl sulfate–polyacrylamide gel electrophoresis (SDS-PAGE) analysis showing expression decarboxylases. Fig. S3. The expression of K3H (a), FjdA and CpdA (b), SrdA and CadA c in western blot analysis. Fig. S4. Hydroxylysine a and 2-OH-PDA b by LC-Q-TOF-MS analysis. [file 12934_2022_1864_MOESM1_ESM.docx]

**Additional file 1**

**Efficient and scalable synthesis of 1,5-diamino-2-hydroxy-pentane from _L_ -lysine via cascade catalysis using engineered *Escherichia coli***

Yangyang Li^1,2^, Alei Zhang^1,2^, Shewei Hu^1,2^, Kequan Chen^1*^, Pingkai Ouyang^1^

^1^College of Biotechnology and Pharmaceutical Engineering, Nanjing Tech University, Nanjing 211816, China

^2^State Key Laboratory of Materials-Oriented Chemical Engineering, Nanjing Tech University, Nanjing 211816, China

*Corresponding author.

E-mail: [kqchen@njtech.edu.cn](mailto:kqchen@njtech.edu.cn).

Tel.: +86-138-1418-0652

**Chemicals**

Tryptone and yeast extract were purchased from Oxoid. Ltd (Hampshire, UK). NaCl, Fmoc chloride, and _L_ -lysine were purchased from Shanghai Aladdin Bio-Chem Technology Co., Ltd (Shanghai, China). Agar-agar powder, _L_-ascorbic acid (Vc), α-ketoglutaric acid, NaH_2_PO_4_·2H_2_O, Na_2_HPO_4_·12H_2_O, NaOH, FeSO_4_·7H_2_O, MgSO_4_·7H_2_O, and D (+)-glucose were purchased from Sinopharm Chemical Reagent Co., Ltd (Shanghai, China). Pyridoxal- 5'-phosphate monohydrate (PLP) was purchased from Shanghai Macklin Biochemical Co., Ltd (Shanghai, China).

**Expression, purification and activity assay of hydroxylase and decarboxylase**

Individual colonies of engineered *E. coli* BL21 (DE3) harboring the above-mentioned recombinant plasmids were cultured in LB medium containing 50 µg/mL kanamycin overnight at 37°C with shaking at 200 rpm. Next, aliquots of the cultures were inoculated into 100 mL of fresh LB medium supplemented with antibiotics in 500 mL flask and incubated at 37°C. Once the cell density (OD_600_) reached 0.6- 0.8, isopropyl β-D-1-thiogalactopyranoside (IPTG) was added to the culture and incubated at 25°C with shaking at 200 rpm for 20­24 h.

Stored cells were resuspended in 50 mM PBS (pH 7.0) and disrupted by sonication, and the cell debris was removed by centrifugation at 8228 × g for 30 min at 4°C. Proteins were initially purified on a Ni-NTA affinity column and then on a HisTrap HP affinity column using an AKTA express system (AKTA Pure 29-0148-30, Sweden). The proteins were eluted with 500 mM imidazole buffer at a flow rate of 3.0 mL·min^−1^. Then, the purified fractions were exchanged into low salt buffer [50 mM PBS (pH 6.0), 5 mM DL-dithiothreitol, and 5% glycerol] using disposable PD-10 desalting columns (Millipore, Burlington, MA, USA).

The hydroxylase activity was analyzed in a reaction mixture containing 200 mg/L K3H enzyme, 20 mM _L_-lysine·HCL, 30 mM α-ketoglutaric acid, 1 mM Vc sodium salt and 1 mM FeSO_4_ in 200µL PBS (50 mM, pH 7.0), at 30°C for 2 h. The activity of decarboxylase was analyzed in a reaction mixture containing 20 mg/L purified enzyme, 40 mM hydroxylysine, and 0.1 mM PLP in 200 µL PBS (50 mM, pH 7.0), at 30°C for 30 min. The samples were heated at 95°C for 10 min, then clarified by centrifugation at 13,523 × *g* for 10 min at room temperature, and the supernatants were used for HPLC detection. One unit of enzyme activity was defined as the amount of enzyme required to consume 1 mmol substrate per min under the described assay conditions.

**The effect of reaction conditions on the whole-cell biocatalysis**

Washed cells were resuspended in 20 mL of 50 mM PBS at varying pH values (4.0, 5.0, 6.0, 7.0, 8.0, and 9.0). The hydroxylysine synthesis reaction contained _L_ -lysine (30, 60, 150, and 250 g/L), α-ketoglutaric acid (molar ratio, 0.5:1, 1:1, 1.5:1, 2:1, and 3:1), Vc (0, 5, 10, 20, and 30 mM), FeSO_4_ (1, 5, 10, 20, and 30 mM), and 5 g dry cell weight (DCW)/L, and was incubated at different temperature (18, 25, 30, 37, and 50°C) with shaking at 200 rpm for different lengths of time (0, 12, 24, 48, and 72 h).

The 2-OH-PDA synthesis reaction contained hydroxylysine (10, 30, 50, and 70 g/L), PLP (0, 0.05, 0.1, and 0.2 mM), and 5 g DCW/L, and was incubated at different pH values (4.0, 5.0, 6.0, 7.0, 8.0, and 9.0) and temperature (20, 30, 40, 50, 60, 70, and 80°C) with shaking at 200 rpm for different lengths of time (0, 6, 12, 24, and 48 h). Samples (0.3 mL) were collected every few hours. Cells were removed by centrifugation at 13,523 × *g* at room temperature for 1 min, and the resulting supernatants were heated at 95°C for 10 min and then clarified by centrifugation at 13,523×*g* for 10 min at room temperature.

**High density fermentation for the engineering strains**

After achieving high concentration cells in the 5-L fermenter, aliquots of the cultures were inoculated into 3 L fresh induction medium. When the OD_600_ reached 2.0, IPTG was added to the culture medium and incubated at 25°C for 20­24 h. During the whole fermentation process, glycerol was fed gradually and maintained below 10 g/L in the culture medium; dissolved oxygen was controlled at 20­30%, and pH was controlled at 6.0­7.0 with ammonia and phosphoric acid. The recombinant *E. coli* cells cultured for 24 h in shaking flasks or in the 5-L fermenter were harvested by centrifugation at 8228 × *g* for 6 min at 4°C. The cell pellets were washed twice with 50 mM potassium phosphate buffer (PBS, pH 7.0) in preparation for the assay or stored at 4°C for later use.

**Analytical methods**

All recombinant protein samples were analyzed by 10% reductive sodium dodecyl sulfate polyacrylamide gel electrophoresis (SDS-PAGE) with 20 mM β-mercaptoethanol incubation. A premixed protein marker (Takara Biotechnology Co., Ltd., Nanjing, China) containing 180-, 140-, 100-, 75-, 60-, and 45-kDa protein bands was used as the molecular mass standard (Supplementary Fig. S1, S2). Protein concentrations were determined by absorption at 595 nm using the Bradford method with bovine serum albumin as the standard [1].

Proteins were analyzed by Western Blot method according to a previous study with modifications [2]. Proteins extracted from cells were separated by 15.0% SDS-PAGE and then transferred to polyvinylidene fluoride (PVDF) membranes. After the membranes were blocked in 5% in BSA buffer for 1 h, the blocking buffer was removed and the membranes were washed four times in 1xPBST buffer (1xPBS buffer+0.1% Tween-20). The membranes were incubated with His tag monoclonal antibody (Invitrogen, 1:1000) at 37°C followed by incubation with a HRP-conjugated goat anti-mouse IG secondary antibody (Invitrogen, 1:10000) for 1 hour. Removing incubation buffer, the membranes were washed four times in 1xPBST buffer and then put into PBS buffer. The resulting bands were detected using super ECL reagent (Fig. S3).

_L_-lysine, hydroxylysine, and 2-OH-PDA concentrations were determined according to a previous study with modifications [3, 4]. The reaction products were treated with 9-fluorenylmethoxycarbonyl chloride (Fmoc-Cl) using the precolumn derivatization method prior to HPLC on an Agilent 1260 Infinity LC system (Agilent Technologies, Santa Clara, CA, USA) equipped with an ultraviolet detector reading at 263 nm and liquid chromatography quadrupole time-of-flight mass spectrometry (LC-Q-TOF-MS) analysis (Supplementary Fig. S4). Separation of samples was achieved using a reverse phase Agilent TC-C18 column (5 × 4.6 mm × 250 mm; Agilent) with a gradient flow rate of 1 mL/min at 40℃ and an injection volume of 10 μL. The mobile phase comprised A (acetonitrile, 0.1% trifluoroacetic acid) and B (water, 0.1% trifluoroacetic acid) and the gradient-elution conditions were as follows: 0 min, 50% A; 8 min, 10% A; 15 min, 10% A;16 min, 50% A; and 21 min, 50% A. The concentration and purity of hydroxylysine and 2-OH-PDA were measured against hydroxylysine and 2-OH-PDA (self-made) standards, respectively, using a calibration curve.

**Table S1** Strains and plasmids used in this work

| Strains or plasmids | Description | Sources |
| --- | --- | --- |
| Strains |  |  |
| Top10 | F- ψ80dlacZ△M15  △(lacZYAargF)U169 DEOr recA1 endA1 hsdR17(rk-mk+)phoA supE44 λ- thi-1 gyrA96 relA1 | General Biosystems |
| BL21(DE3) | F- ompT gal dcm lon hsdSb(Rb-Mb-) λ(DE3 [lacI lac lacUV5-T7 gene 1 ind1 sam7 nin5]) | General Biosystems |
| B1 | BL21(DE3) carrying pRSFDuet1-*K3H* | This work |
| B2 | BL21(DE3) carrying pRSFDuet1- *K3H-lysP* | This work |
| B3 | BL21(DE3) carrying pRSFDuet1-*K3H*-*YbjE* | This work |
| B4 | BL21(DE3) carrying pRSFDuet1- *K3H-HisQ* | This work |
| B5 | BL21(DE3) carrying pRSFDuet1- *K3H-HisM* | This work |
| B6 | BL21(DE3) carrying pRSFDuet1-*K3H*-*HisP* | This work |
| B7 | BL21(DE3) carrying pRSFDuet1-*K3H*-*argO* | This work |
| B8 | BL21(DE3) carrying pRSFDuet1- *K3H-cadB* | This work |
| B9 | BL21(DE3) carrying pRSFDuet1- *K3H-argT* | This work |
| B10 | BL21(DE3) carrying pRSFDuet1-*K3H*-*lysP*-*YbjE* | This work |
| B11 | BL21(DE3) carrying pRSFDuet1-*K3H*-*lysP*-*argO* | This work |
| B12 | BL21(DE3) carrying pRSFDuet1-*K3H*-*CadB*-*YbjE* | This work |
| B13 | BL21(DE3) carrying pRSFDuet1-*K3H*-*CadB*-*argO* | This work |
| B14 | BL21(DE3) carrying pRSFDuet1-*K3H*-*CadB*-*argT* | This work |
| B15 | BL21(DE3) carrying pRSFDuet1-*CadA* | This work |
| B16 | BL21(DE3) carrying pRSFDuet1-*CpdA* | This work |
| B17 | BL21(DE3) carrying pRSFDuet1-*SrdA* | This work |
| B18 | BL21(DE3) carrying pRSFDuet1-*FjdA* | This work |
| pRSFDuet1 | *ColE1* ori, KanR, *E. coli* expression vector | This work |

**Table S2** Primers used in this work

| Primer | Sequence(5’-3’) |
| --- | --- |
| CadB-F | GA*agatct*GATGAGTTCTGCCAAGAAGATCGG |
| CadB-R | CCG*ctcgag*TTAATGTGCGTTAGACGCGGTG |
| YbjE-F | GA*agatct*GATGTTTTCTGGGCTGTTAATC |
| YbjE-R  HisM-F  HisM-R  HisQ-F  HisQ-R  HisP-F  HisP-R  argT-F  argT-R  argO-F  argO-R  lysP-F  lysP-R  CBYE-F  CBYE-R  CBaO-F  LPaO-R  CBaT-F | CCG*ctcgag*TTACGCAGAGAAAAAGGCGATG  GA*agatct*GATGATCGAAATCTTACATGAATACTGG  CCG*ctcgag*TCAGTGCGTTGAAGAAGGTTTCAC  GA*agatct*GATGTTGTATGGGTTTTCAGGTG  CCG*ctcgag*TCACAGGTCAGCCCTCTTCAC  GA*agatct*GATGTCCGAGAATAAATTAAACG  CCG*ctcgag*TTATTTCAGCGATCCCTTAAGG  GA*agatct*GATGAAGAAGTCGATTCTCGCTC  CCG*ctcgag*TCAGTCACCGTAGACATTAAAGTC  GA*agatct*GATGTTTTCTTATTACTTTCAAGGTC  CCG*ctcgag*CTAACTGAACAAGGCTTGTGC  GA*agatct*GATGGTTTCCGAAACTAAAACCACAG  CCG*ctcgag*TTATTTCTTATCGTTCTGCGGG  *gaattc***AAGGAG**atata*gatatc*ATGTTTTCTGGGCTGTTAATC  *gatatc*tatat**CTCCTT***gaattc*TTAATGTGCGTTAGACGCGG  *gaattc***AAGGAG**atata*gatatc*ATGTTTTCTTATTACTTTCAAGGTC  *gatatc*tatat**CTCCTT***gaattc*TTATTTCTTATCGTTCTGCG  *gaattc***AAGGAG**atata*gatatc*ATGAAGAAGTCGATTCTCGC |
|  |  |

Restriction endonuclease-lowercase italic letters, RBS-capital black letters, spacer sequences between RBS and restriction endonuclease- lowercase letters.





**Fig. S1.** Sodium dodecyl sulfate–polyacrylamide gel electrophoresis (SDS-PAGE) analysis showing expression _L_ -lysine 3-hydroxylase K3H

Notes: M-protein maker, 1- soluble (background expression，incubation 25℃), 2- insolube (background expression, incubation 25℃), 3- soluble K3H (37.78kDa，incubation 15℃), 4- insolube (incubation 15℃), 5- soluble K3H (incubation 20℃), 6- insoluble K3H (incubation 20℃), 7- solube K3H (incubation 25℃), 8- insolube K3H (incubation 25℃), 9- solube K3H (incubation 30℃), 10- insolube K3H (incubation 30℃), 11- pure enzyme K3H.





**Fig. S2.** Sodium dodecyl sulfate–polyacrylamide gel electrophoresis (SDS-PAGE) analysis showing expression decarboxylases

Notes: M-protein maker, 1- soluble (background expression), 2- insolube (background expression), 3- soluble FjdA (57.05kDa), 4- insolube FjdA, 5- soluble CpdA (56.45kDa), 6- insoluble CpdA, 7- solube SrdA (43.22kDa), 8- insolube SrdA, 9- solube CadA (82.47kDa), 10- insolube CadA, 11- pure enzyme FjdA, 12- pure enzyme CpdA, 13- pure enzyme SrdA, 14- pure enzyme CadA.





**Fig. S3.** The expression of K3H (a), FjdA and CpdA (b), SrdA and CadA (c) in western blot analysis

1. K3H- _L_-lysine 3-hydroxylase, 37.78kDa; (b) FjdA- hydroxylysine decarboxylase, 57.05kDa; CpdA- hydroxylysine decarboxylase, 56.45kDa; (c) SrdA- hydroxylysine decarboxylase, 43.22kDa; CadA- hydroxylysine decarboxylase, 82.47kDa

Notes:

a) M-protein maker, 1- positive control, 2- soluble (background expression), 3- insoluble (background expression), 4- soluble (K3H), 5- insoluble (K3H);

b) M-protein maker, 1- positive control, 2- soluble (background expression), 3- insoluble (background expression), 4- soluble (FjdA), 5- insoluble (FjdA), 6- soluble (background expression), 7- insoluble (background expression), 8- soluble (CpdA), 9- insoluble (CpdA);

c) M- protein maker, 1- positive control, 2- soluble (background expression), 3- insoluble (background expression), 4- soluble (SrdA), 5- insoluble (SrdA), 6- soluble (background expression), 7- insoluble (background expression), 8- soluble (CadA), 9- insoluble (CadA).

a)

b)

**Fig. S4.** Hydroxylysine (a) and 2-OH-PDA (b) by LC-Q-TOF-MS analysis

**References**

1. Bradford MM: **A rapid and sensitive method for the quantitation of microgram quantities of protein utilizing the principle of protein-dye binding.** *Analytical biochemistry* 1976, **72:**248-254.

2. Liu J, Lin J, He S, Wu C, Wang B, Liu J, Duan Y, Liu T, Shan S, Yang K, et al: **Transgenic Overexpression of IL-37 Protects Against Atherosclerosis and Strengthens Plaque Stability.** *Cellular Physiology and Biochemistry* 2018, 45:1034-1050.

3. Baud D, Peruch O, Saaidi PL, Fossey A, Mariage A, Petit JL, Salanoubat M, Vergne-Vaxelaire C, de Berardinis V, Zaparucha A: **Biocatalytic approaches towards the synthesis of chiral amino alcohols from lysine: cascade reactions combining alpha-keto acid oxygenase hydroxylation with pyridoxal phosphate- dependent decarboxylation.** *Advanced Synthesis & Catalysis* 2017, **359:**1563-1569.

4. Hu S, Yang P, Li Y, Zhang A, Chen K, Ouyang P: **Biosynthesis of cis-3-hydroxypipecolic acid from _L_ -lysine using an in vivo dual-enzyme cascade.** *Enzyme and microbial technology* 2022, **154:**109958-109958.
